# Arthur's Method of Treating Dental Caries

**Published:** 1872-11

**Authors:** S. G. Perry

**Affiliations:** Secretary of Odontological Society of New York.


### ARTICLE V.

### *Arthur's Method of Treating Dental Caries.*

Dr. Robert Arthur, being received with applause, said:

*Gentlemen:*—I cannot but feel gratified and flattered at this cordial reception. I am here this evening at the invitation of the Odontological Society of New York, to make some further statements than appear in my recent publication, in regard to the method of treating the teeth in reference to dental caries, therein explained.

Innovators, if their views pass merit, have less to fear from opposition than from indifference. I certainly cannot complain of neglect, in view of the lively and widely extended interest displayed in the system I have proposed. While I have confidently believed that in time this system must be generally adopted, I was not prepared for its immediate acceptance to so great an extent as I have evidence that it has been. Instead of opposition, I have been surprised with the reception it has met, and really somewhat overwhelmed with the expressions of approval I have heard during the few hours I have been in New York.

I shall, of course, not occupy your time this evening in going over the general argument I have attempted in regard to the treatment and prevention of decay. This I have already done very elaborately, and you are familiar with the course of reasoning I have pursued. I shall confine myself, at the present time, to a brief comparison of the method I propose with that now generally relied upon.

As is well understood, the great remedy for dental caries is that of filling the carious cavities with gold. You are all acquainted with the remarkable improvements which have, within the past fifteen years, been made in gold prepared for dental purposes, and in the instruments and appliances of all kinds for the operation in question. Results not dreamed of twenty years ago are now attainable, and the number of "good operators," as compared with the aggregate of those engaged in the practice of dentistry, has greatly increased.

But it must be admitted that while gold is by far the best material now known for the purpose, it has some serious defects. The color of gold, differing so widely from the natural hue of the teeth, leads to disfiguration when it is employed at positions where it becomes visible. It is under certain circustances, very difficult of manipulation, and requires a high order of manuel skill to produce beneficial results. Its costliness, and the great length of time required for the performance with it of difficult operations, so frequently required, place the preservation of the teeth by this means out of the reach of people of moderate means.

This latter consideration is one of great importance, and one which we have no right to ignore. It is incumbent upon every true man engaged in the practice of a profession involving the health and comfort of the community, as is the case in an eminent degree with ours, to do all in his power, consistently with his just demand for full compensation for his services, to bring its benefits within the reach of as large a number of the community as possible.

I need not inform you that great and persistent efforts have been made to discover a substitute for gold for dental operations, and that a variety of plastic substances or compounds, some of them resembling the natural hue of the teeth, have been invented. But no one will pretend to say that they have begun to displace gold in the hands of the best practitioners.

But it seems to have been overlooked that the solution of the problem of the treatment of dental caries, when it can be restored to a a sufficiently early age, lies in another direction. The dental profession seems to have lost sight of the numerous examples of the successful treatment of incipient dental caries of the proximate surfaces of the teeth furnished by the practice of Hayden, Parmly, and others, by its removal with the file. It seems not to have occurred to the most earnest men that if incipient caries should be arrested by this plan, it might by the same means be prevented from occurring in cases where it was otherwise inevitable. This is all that I propose. This is the solution of the problem which I have offered; and I propose now to call your attention to some of the results of this method of practice, and to compare them with those usually relied upon. I will not take cases of other practitioners, in order to how their failures in comparison with the success of my own treatment; but, for illustration, I will take two cases which I have myself treated by the two methods under consideration. Whatever in the way of failure of good results may appear, the responsibility must rest entirely with myself.

[Dr. A. here exhibited enlarged diagrams, showing all the operations in cases presented for illustration.]

It has been objected to the practice I propose, that while it might be applicable to well formed teeth in which caries progresses slowly, it would fail when applied to frail teeth.

It has been said that the success of the older practitioners in arresting caries by the use of the file was due to the better quality of teeth in their day—that the teeth have deteriorated so much at the present time that it would fail if attempted.

The cases I have selected for illustration will show the fallacy of this assumption. The two individuals are members of the same family, a sister and brother. Their physical characteristics, and the structure of their teeth are closely similar. These cases, it must be admitted, furnish a test of the fairest description, and afford ground for a just comparison of the merits of both methods of practice.

The treatment of these cases was commenced in 1862. The girl was then fifteen years of age; the boy eight. They are under my professional care at the present time.

In the case of the young lady, caries had attacked the proximate surface of all the teeth except the lower incisors, and had progressed, with a small number of exceptions, so far as to render filling necessary.

Where the caries was in its incipient stage it was removed. and the affected teeth permanently separated. The proximate fillings amounted in number to forty-seven. Incipient caries was removed from six surfaces. The operation in this case, when fillings were required were extremely painful, as the dentine was highly sensitive. Notwithstanding the care with which the fillings were inserted, it became necessary to remove some of them several times, in consequence of a recurrence of caries at some point about the margins of the fillings. There was no recurrence of caries in a single instance where the teeth were treated for incipient caries and permanently separated.

In the case of the boy, the course of treatment I have proposed was rigidly carried out. He is now eighteen years of age. A recent examination shows thirty-one proximate surfaces treated as I advised. In six, caries recurred and fillings were required. These were points at which caries had occured, and was not, probably, entirely removed.

With the exception of the fillings, the operations were painless.

Let us now consider the prominent features of these cases. The teeth of two individuals were equally liable to decay. In the case of the sister, caries had attacked every proximate surface, with the exceptions noted, before she had reached the age of fifteen.

What reason was there to suppose, as the conditions were nearly identical, that the teeth of the brother would not suffer in the same manner? In his case the occurrence of caries was prevented in the manner I have proposed, except in a small number of instances. These were the only teeth treated as described in which there was a recurrence of caries. In the case of the sister, although I operated with all the care I was capable of exercising, I found it necessary to refill some of the bicuspid and molar teeth several times, in consequenc of the recurrence of caries. But the six surfaces from which incipient caries had been removed at least eight years ago still remain unchanged. If this case alone was taken as a test, who could dispute the advantages of the treatment employed?

Now, these are not special cases selected for this occasion. I had indeed prepared diagrams of others, but they were made where I could not direct the artist, and do not well represent what I wish to show. But it is not important. The cases to which I have called attention represents the results of my practice for the last ten years. During this period I have pursued this course with all children, with rare exceptions (for the cases in which there is exception from caries of all proximate surfaces, except those of the lower front teeth, are at the present day very rare) who have come under my professional care. I now see them frequently as young men and women, with their teeth in such condition as to require no further treatment of consequence, at an age when for most individuals commence the serious of painful operations which are to be continued from year to year during a lifetime, and which, in the hands of many dentists, prove frequently unsuccessful.

Now, in the name of humanity, what man here present would subject his children—and his patients occupy the same relation to him professionally—to the agonizing treatment required in the first case, when the more effective and painless treatment pursued in the second could be applied?

I beg to say, gentlemen, that I have not rashly come to my present conclusions. I have given my earnest attention to this subject for more than twenty years. So cautiously have I proceeded, in view of the adverse opinions and predjudices of my professional brethern, that with my convictions of what I believed to be correct practice, it seems to me like stupidity, looking back upon my past career, that I did not adopt the practice fully long ago.

I am aware that it has been proposed to prevent caries by great personal care. While I am satisfied that this might, in some cases be accomplished, it has proved unsuccessful in my own hands. I should be glad to resort invariably to this plan if I could make it available.

In attempting this practice, it is important that, if possible, it should be strictly preventive. If caries be permitted to progress so far as to attack the dentine, it is exceedingly difficult to remove it perfectly, and most of the cases of failure in my own hands, and in those of others, have been where this has occurred. Every dentist who deserves the name should be able to determine when caries of the proximate surfaces of the bicuspids and molar teeth is inevitable unless prevented.

The complaint of inconvenience in the act of mastication is in those cases where caries has been allowed to progress so far as to require the separation to be wider than is necessary, if the treatment is conducted in the manner I have advised. I have never heard complaint from this cause, as a means of prevention of caries, in carrying out my method.

The best method of making these separations is a matter that will soon be determined in a better manner, I presume, than I have explained, or can explain at the present time. The separations, in the case referred to, were made with the file. I am well aware of the reasonable objections to the employment of this instrument. I have suffered from this cause, and nothing but a severe sense of duty could have sustained me in my determination to carry inflexibly into practice the system I knew to be best for those who intrusted themselves and their children to my care. With the greater part of my profession in my own vicinity misunderstanding, and arrayed against, the practice, it came to be generally understood that I proposed the indiscriminate filling apart of all the teeth of all the children who were so unfortunate as to fall into my hands.

But for some time past I have abandoned the use of the file, and employed the hard chisels described in my late work. With them the object can be well effected. The process, I must admit, is tedious and laborious as applied to the molar teeth, but is much less objectionable to the patients, and for this reason is greatly preferable to the file.

The introduction of Morrison's engine and other appliances of this description, however, opens a new field for improvement in this way, and I have no question that means will be employed which will greatly facilitate the operation of separating the teeth. I expected to be able to exhibit to you specimens of instruments which I have recently devised for the purpose, and which I am sure will do much toward removing difficulties now attending this treatment; but I have failed to receive them I have no question that still other improvements will be made, which will supersede any means now relied upon. With the present facilities, the cone bur drills may be used with excellent effect in connection with the chisels described for separating the molars, and what are known as " fissure burs," the bicuspid and incisor and also the molar teeth.

It has now been some six years since I began to bring this subject to the attention of the dental profession. have challenged discussion, and hold myself ready at all times to defend my position. During this time I have not met with a single objection of any force or weight to the practice proposed. But, as it is growing late, and other subjects of importance will no doubt be brought to your attention, I will not detain you longer.—*Report of* S. G. PERRY, *Secretary of Odontological Society of New York.*

---

